# Levenberg-Marquardt Method for the Eigenvalue Complementarity Problem

**DOI:** 10.1155/2014/307823

**Published:** 2014-10-30

**Authors:** Yuan-yuan Chen, Yan Gao

**Affiliations:** ^1^School of Management, University of Shanghai for Science and Technology, Shanghai 200093, China; ^2^College of Mathematics, Qingdao University, Qingdao 266071, China

## Abstract

The eigenvalue complementarity problem (EiCP) is a kind of very useful model, which is widely used in the study of many problems in mechanics, engineering, and economics. The EiCP was shown to be equivalent to a special nonlinear complementarity problem or a mathematical programming problem with complementarity constraints. The existing methods for solving the EiCP are all nonsmooth methods, including nonsmooth or semismooth Newton type methods. In this paper, we reformulate the EiCP as a system of continuously differentiable equations and give the Levenberg-Marquardt method to solve them. Under mild assumptions, the method is proved globally convergent. Finally, some numerical results and the extensions of the method are also given. The numerical experiments highlight the efficiency of the method.

## 1. Introduction

Eigenvalue complementarity problem (EiCP) is proposed in the study of the problems in mechanics, engineering, and economics. The EiCP is also called cone-constrained eigenvalue problem in [1–4]. The EiCP is to find a solution including a scalar and a nonzero vector satisfying a complementarity constraint on a closed convex cone. The EiCP can be reformulated to be a special complementarity problem or a mathematical programming optimization problem with complementarity constraints and can use nonsmooth or semismooth Newton type method to solve it, such as [5–7]. The Levenberg-Marquardt method is one of the widely used methods in solving optimization problems (see, for instance, [8–15]). Use a trust region strategy to replace the line search, the Levenberg-Marquardt method is widely considered to be the progenitor of the trust region method approach for general unconstrained or constrained optimization problems. The use of a trust region avoids the weaknesses of Gauss Newton method, that is, its behavior when the Jacobian is rank deficient or nearly so rank deficient. On the other hand, we reformulate the EiCP as a system of continuously differentiable equations that is one of the most interesting themes. The advantage of the reformulation is that we solve the equations with continuously differentiable functions for which there are rich powerful solution methods and theory analysis, including the powerful Levenberg-Marquardt method. So, in this paper, we give the Levenberg-Marquardt method to solve the EiCP. The EiCP, which we will consider, is the following problem. Given the matrix *A* ∈ *R* ^*n*×*n*^ and the matrix *B* ∈ *R* ^*n*×*n*^, which are positive definite matrix, then we consider to find a scalar *λ* ∈ *R* and a vector *x* ∈ *R* ^*n*^∖{0}, such that

(1) $$\begin{array}{c} \left(A-\lambda B\right)x\geq 0,\\ x\geq 0,\\ x^{T}\left(A-\lambda B\right)x=0. \end{array}$$

This paper is organized as follows. In Section 2, we give some background definitions and known properties. And we also give the Levenberg-Marquardt method for the EiCP. The global convergence analysis and some discussions of the Levenberg-Marquardt method is also given. In Sections 3 and 4, we give some numerical results and some extensions of the method.

Throughout the paper, *M*_*n*_(*R*) denotes a real matrix of order *n* and *M*_*n*,*m*_(*R*) denotes a real matrix of order *n* × *m*. 0_*n*_ = (0,…, 0) and *e*_*n*_ = (1,…, 1).

## 2. Preliminaries

In this section, firstly, we reformulate (1) as a system of continuously differentiable equations and give some preliminaries used in the following. Then, we propose the Levenberg-Marquardt method for the EiCP.

As we all know, (1) can be rewritten as

(2) ω=xλ,f(ω)=In,0ω=x,g(ω)=A,0ω−0n,1ωB,0ω=(A−λB)x,fTωgω=0,en,0ω=1,

where *f* : *R* ^*n*+1^ → *R* ^*n*^ is a continuously differentiable function and the matrix *A* ∈ *R* ^*n*×*n*^. Using the nonlinear complementarity problem (NCP) function *ϕ* : *R* ^2^ → *R*, which satisfied the following basic property

(3) $$\begin{array}{c} \phi \left(a,b\right)=0\;\text{iff}\;\;ab=0,\;\;a\geq 0,\;\;b\geq 0. \end{array}$$

By the property, (2) can be recasted as the following system of equations:

(4) $$\begin{array}{c} H\left(\omega \right)=\left(\begin{array}{c} H_{1}\left(\omega \right)\\ \vdots \\ H_{n}\left(\omega \right) \end{array}\right)=\left(\begin{array}{c} \phi \left(f_{1}\left(\omega \right),g_{1}\left(\omega \right)\right)\\ \vdots \\ \phi \left(f_{n}\left(\omega \right),g_{n}\left(\omega \right)\right) \end{array}\right)=0,\\ \left(e_{n},0\right)\omega =1. \end{array}$$

Then, *ω* solves the EiCP (1) if and only if *ω* solves (4). Define a merit function for (4) as

(5) $$\begin{array}{c} \Psi \left(\omega \right)=\frac{1}{2}\overset{n}{\underset{i=1}{\sum }}\phi^{2}\left(f_{i}\left(\omega \right),g_{i}\left(\omega \right)\right)=\frac{1}{2}{||H\left(\omega \right)||}^{2}. \end{array}$$

We use a special NCP function named Fischer-Burmeister function, defined as

(6) $$\begin{array}{c} \phi \left(a,b\right)=\sqrt{a^{2}+b^{2}}-\left(a+b\right). \end{array}$$

We know that the favorable property of Ψ is that Ψ is a continuously differentiable function on the whole space, although *H* is not a continuously differentiable function in general (see, for example, [16]). Thus, we give the system of continuously differentiable equations for (1) as the following equations:

(7) $$\begin{array}{c} F\left(\omega \right)=\left(\begin{array}{c} \Psi \left(\omega \right)\\ \left(e_{n},0\right)\omega -1 \end{array}\right)=0, \end{array}$$

where *F* : *R* ^*n*+1^ → *R* ^2^, which is a continuously differentiable function. In what follows, we will give the Levenberg-Marquardt method. Denote

(8) $$\begin{array}{c} \Phi \left(\omega \right)=\frac{1}{2}{||F\left(\omega \right)||}^{2}. \end{array}$$

The least-squares formulation of (7) is the following unconstrained optimization problem:

(9) $$\begin{array}{c} \underset{\omega \in R^{n+1}}{\min}\Phi \left(\omega \right)=\frac{1}{2}{||F(\omega )||}^{2}. \end{array}$$

Now, we give the Levenberg-Marquardt method for solving (1). The global convergence result of the method is also given.

**Method 1 (the Levenberg-Marquardt method for the EiCP).**

Given 0 < *α* < 1, 0 < *β* < 1, *p* > 2, *ρ* > 0, 0 < *δ* ≤ 2. *P* > 0, *ϵ* > 0, *ω*_0_ ∈ *R* ^*n*+1^, *μ*_0_ = ||*F*(*ω*_0_)||^*δ*^, and *k* : = 0.

**Step 1 .**

If ||∇Φ(*ω*)||≤*ϵ*, then stop. Otherwise, compute *d*_*k*_ by

(10) $$\begin{array}{c} \left(F^{\text{'}}{\left(\omega_{k}\right)}^{T}F^{\text{'}}\left(\omega_{k}\right)+\mu_{k}I\right)d+F^{\text{'}}{\left(\omega_{k}\right)}^{T}F\left(\omega_{k}\right)=0. \end{array}$$

**Step 2 .**

If ∇Φ(*ω*_*k*_)^*T*^ *d*_*k*_ + *ρ*||*d*_*k*_||^*P*^ > 0, let *d*_*k*_ = −∇Φ(*ω*_*k*_); otherwise, *d*_*k*_ is computed by (10). Then, find the smallest nonnegative integer *m* such that

(11) $$\begin{array}{c} \Phi \left(\omega_{k}+\beta^{m}d_{k}\right)\leq \Phi \left(\omega_{k}\right)+\alpha \beta^{m}\nabla \Phi {\left(\omega_{k}\right)}^{T}d_{k}. \end{array}$$

Set *ω*_*k*+1_ = *ω*_*k*_ + *β* ^*m*^ *d*_*k*_.

**Step 3 .**

Let *μ*_*k*+1_ = ||*F*(*ω*_*k*+1_)||^*δ*^ and *k* : = *k* + 1, and go to Step 1.

Now, we give the global convergence of Method 1.

**Theorem 1 .**

Suppose that {*ω*_*k*_}, *k* = 1,2,… generated by Method 1. Then, each accumulation point of the sequence is a stationary point of Φ.

**Proof**

Suppose that {*ω*_*k*_}_*K*_ → *ω* ^⋆^, {*ω*_*k*_}_*K*_ is a subsequence of {*ω*_*k*_}, and *k* = 1,2,…. When there are infinitely many *k* ∈ *K* such that *d*_*k*_ = −∇Φ(*ω*_*k*_), by Proposition  1.16 in [17], we get the assertion. In the following, we assume that if {*ω*_*k*_}_*K*_ is a convergent subsequence of {*ω*_*k*_}, then *d*_*k*_ is always computed by (10). We assume that for every convergent subsequence {*ω*_*k*_}_*K*_ for which

(12) $$\begin{array}{c} \underset{k\in \mathrm{Kk}\to \infty }{\lim}\nabla \Phi \left(\omega_{k}\right)\neq 0, \end{array}$$

we have

(13) $$\begin{array}{c} \underset{k\in Kk\to \infty }{\lim\;\sup}||d_{k}||<\infty , \end{array}$$

(14) $$\begin{array}{c} \underset{k\in Kk\to \infty }{\lim\;\sup}\left|\nabla \Phi {\left(\omega_{k}\right)}^{T}d_{k}\right|>0. \end{array}$$

In the following, we also assume that *ω*_*k*_ → *ω* ^⋆^. Suppose that *ω* ^⋆^ is not a stationary point of Φ. By (10), we have

(15) ∇Φ(ωk)=F'ωkTF'ωk+μkIdk≤F'ωkTF'ωk+μkIdk;

therefore, we have

(16) $$\begin{array}{c} ||d_{k}||\geq \frac{||\nabla \Phi \left(\omega_{k}\right)||}{||\left(F^{\text{'}}{\left(\omega_{k}\right)}^{T}F^{\text{'}}\left(\omega_{k}\right)+\mu_{k}I\right)||}. \end{array}$$

Obviously, the denominator in the above inequality is nonzero; otherwise, we have ||∇Φ(*ω*_*k*_)|| = 0. Then, the algorithm has stopped. On the other hand, we know that there exists a constant *ζ* > 0 such that

(17) $$\begin{array}{c} ||F^{\text{'}}{\left(\omega_{k}\right)}^{T}F^{\text{'}}\left(\omega_{k}\right)+\mu_{k}I||\leq \zeta ; \end{array}$$

moreover,

(18) $$\begin{array}{c} ||d_{k}||\geq \frac{||\nabla \Phi \left(\omega_{k}\right)||}{\zeta }. \end{array}$$

By ∇Φ(*ω*_*k*_)^*T*^ *d*_*k*_ ≤ −*ρ*||*d*_*k*_||^*P*^ and the fact that the gradient ∇Φ(*ω*_*k*_) is bounded on the convergent sequence {*ω*_*k*_}, we get (13). We next prove (14). If (14) is not satisfied, there exists a subsequence {*ω*_*k*_}_*K*′_ of {*ω*_*k*_}_*K*_:

(19) $$\begin{array}{c} \underset{k\in K^{\text{'}}k\to \infty }{\lim}|\nabla \Phi {\left(\omega_{k}\right)}^{T}d_{k}|=0. \end{array}$$

This implies that lim⁡_*k*∈*K*′*k*→*∞*_||*d*_*k*_|| = 0. From (18), we know that

(20) $$\begin{array}{c} \underset{k\in K^{\text{'}}k\to \infty }{\lim}||\nabla \Phi \left(\omega_{k}\right)||=0, \end{array}$$

which contradicts with (12). Thus, (14) holds. So, according to the definition given in [17], the sequence {*d*_*k*_} is uniformly gradient related to {*ω*_*k*_}. We complete the proof.

**Remark 2 .**

In Method 1, we can also use some other line search, such as the nonmonotone line search. The line search is to find the smallest nonnegative integer *m* such that

(21) $$\begin{array}{c} \Phi \left(\omega_{k}+\beta^{m}d_{k}\right)-\underset{0\leq j\leq m\left(k\right)}{\max}\Phi \left(\omega_{k-j}\right)-\alpha \beta^{m}\nabla \Phi {\left(\omega_{k}\right)}^{T}d_{k}\leq 0, \end{array}$$

where *m*(0) = 0, *m*(*k*) = min⁡{*M*_0_, *m*(*k* − 1) + 1}, and *M*_0_ > 0 is a integer.

**Remark 3 .**

In Method 1, we can also use the following equation to compute *d*_*k*_ in Step 1. We can find an approximate solution *d*_*k*_ ∈ *R* ^*n*^ of the equation

(22) $$\begin{array}{c} \left(F^{\text{'}}{\left(\omega_{k}\right)}^{T}F^{\text{'}}\left(\omega_{k}\right)+\mu_{k}I\right)d+F^{\text{'}}{\left(\omega_{k}\right)}^{T}F\left(\omega_{k}\right)-r_{k}=0, \end{array}$$

where *r*_*k*_ is the residuals and satisfies

(23) $$\begin{array}{c} ||r_{k}||\leq \alpha_{k}||\nabla \Phi \left(\omega_{k}\right)||, \end{array}$$

where *α*_*k*_ ≤ *a* < 1 for every *k*.

## 3. Numerical Results

We give some numerical experiments for the method. And we compare Method 1 with the scaling and projection algorithm (denoted by SPA in [18]). The numerical results indicate that Method 1 works quite well in practice. We consider the eigenvalue complementarity problems, which are all taken form [3, 18]. All codes for the method are finished in MATLAB. The parameters used in the method are chosen as *ρ* = 10, *P* = 3, *α* = 0.1, and *ϵ* = 10^−4^.

**Example 4 .**

We consider

(24) $$\begin{array}{c} \left(A-\lambda B\right)x\geq 0,\\ x\geq 0,\\ x^{T}\left(A-\lambda B\right)x=0, \end{array}$$

where

(25) B=1001,A=8−134.

By [3], we know that Example 4 has three eigenvalues. Now, we consider random initial points to compute Example 4 by Method 1. Numerical results for Example 4 by Method 1 and SPA method are presented in Table 1.

Table 1 shows that Method 1 are able to detect all the solutions for the small size matrix. But the SPA method can only detect 2 solutions from [3].

**Example 5 .**

We consider

(26) $$\begin{array}{c} \left(A-\lambda B\right)x\geq 0,\\ x\geq 0,\\ x^{T}\left(A-\lambda B\right)x=0, \end{array}$$

where

(27) B=100010001,A=8−14340.52−0.56.

From [3], we also know that Example 5 have 9 eigenvalues. Now, we consider random initial points to compute the above Example 5 by Method 1. Numerical results for Example 5 by Method 1 and the SPA method are presented in Table 2.

Table 2 shows that Method 1 is able to detect all the solutions. But, the SPA method can only detect 4 Pareto eigenvalues from the analysis of [3].

**Example 6 .**

We consider

(28) $$\begin{array}{c} \left(A-\lambda B\right)x\geq 0,\\ x\geq 0,\\ x^{T}\left(A-\lambda B\right)x=0, \end{array}$$

where

(29) B=100010001,A=100106−18−8192158−24−10124437−7213802.

From [3], we also know that Example 6 have 23 eigenvalues. Now, we consider random initial points to compute Example 6 by Method 1. Numerical results for Example 6 by Method 1 and the SPA method are given in Table 3.

Table 3 shows that Method 1 is able to detect all 23 solutions. But, the SPA method can only detect 2 Pareto eigenvalues from [3]. The numerical results indicate that Method 1 works quite well for the big size EiCP in practice.

*Discussion*. In this section, we study the numerical behaviors of Method 1 for solving the Pareto eigenvalue problem. The EiCP problem is very useful in studying the optimization problems arising in many areas of the applied mathematics and mechanics. By using the F-B function, we reformulate the EiCP as a system of continuously differentiable equations. Then, we use the Levenberg-Marquardt method to solve it. From the above numerical results, we know that Method 1 is very effective for small size and big size EiCP problems.

## 4. Extensions

### 4.1. Bieigenvalue Complementarity Problems (BECP)

We also can use Method 1 to solve the bieigenvalue complementarity problems (denoted by BECP). The BECP is to find (*λ*, *μ*) ∈ *R* × *R* and (*x*, *y*) ∈ *R* ^*n*^∖{0} × *R* ^*m*^∖{0} such that

(30) $$\begin{array}{c} x\geq 0,\;\lambda x-Ax-By\geq 0,\;x^{T}\left(\lambda x-Ax-By\right)=0,\\ y\geq 0,\;\mu y-Cx-Dy\geq 0,\;y^{T}\left(\mu y-Cx-Dy\right)=0, \end{array}$$

where *A* ∈ *M*_*n*_(*R*), *B* ∈ *M*_*n*,*m*_(*R*), *B* ∈ *M*_*m*,*n*_(*R*), and *D* ∈ *M*_*m*_(*R*).

Let $\omega =\left(\begin{array}{c} x\\ y\\ \lambda \\ \mu \end{array}\right)$, *f*(*ω*) = *x*, *g*(*ω*) = *y*, *P*(*ω*) = *λx* − *Ax* − *By*, and *Q*(*ω*) = *μy* − *Cx* − *Dy*. We can write the above bieigenvalue complementarity problems as *f*(*ω*) ≥ 0, *g*(*ω*) ≥ 0, *P*(*ω*) ≥ 0, *Q*(*ω*) ≥ 0, *f* ^*T*^(*ω*)*P*(*ω*) = 0, *g* ^*T*^(*ω*)*Q*(*ω*) = 0, (*e*_*n*_, 0_*n*_, 0,0)*ω* = 1, and (0_*n*_, *e*_*n*_, 0,0)*ω* = 1. By using F-B function, similar to rewriting the EiCP, we can rewrite the above bieigenvalue complementarity problems as the following equations:

(31) H1(ω)=ϕ(f1(ω),P1(ω))⋮ϕ(fn(ω),Pn(ω))=0,H2(ω)=ϕ(g1(ω),Q1(ω))⋮ϕ(gn(ω),Qn(ω))=0,(en,0n,0,0)ω−1=0,(0n,en,0,0)ω−1=0.

Let

(32) Φ1(ω)=12H1(ω)2,Φ2(ω)=12H2(ω)2.

Then, we can give the system of continuously differentiable equations for the bieigenvalue complementarity problems as the following continuously differentiable equations:

(33) $$\begin{array}{c} \left(\begin{array}{c} \Phi_{1}\left(\omega \right)\\ \Phi_{2}\left(\omega \right)\\ \left(e_{n},0_{n},0,0\right)\omega -1\\ \left(0_{n},e_{n},0,0\right)\omega -1 \end{array}\right)=0. \end{array}$$

In the following numerical test, the parameters used in the method are also chosen as *ρ* = 10, *P* = 3, *α* = 0.1, and *ϵ* = 10^−4^.

**Example 7 .**

We consider the BECP, where *A* = 0, *D* = 0,

(34) $$\begin{array}{c} B=\left(\begin{array}{cc} 1 & 0\\ 0 & 1 \end{array}\right),\\ C=\left(\begin{array}{cc} 1 & 0\\ 0 & 1 \end{array}\right). \end{array}$$

We use Method 1 to solve the Example 7. Results for Example 7 with random initial point are given in Table 4.

### 4.2. The Paretian Version

We consider using Method 1 to compute the following Paretian version:

(35) $$\begin{array}{c} x\geq 0,\;M\left(\lambda \right)x\geq 0,\;x^{T}M\left(\lambda \right)x=0, \end{array}$$

where *M* standing for the pencil associated to a finite collection {*A*_0_, *A*_1_,…, *A*_*r*_} of real matrices of order *n* and

(36) $$\begin{array}{c} M\left(\lambda \right)=\overset{r}{\underset{k=0}{\sum }}\lambda^{k}A_{k}. \end{array}$$

**Example 8 .**

We consider the quadratic pencil model, where

(37) $$\begin{array}{c} M\left(\lambda \right)=\lambda^{2}\left(\begin{array}{ccc} 2 & 0 & 0\\ 0 & 6 & 0\\ 0 & 0 & 10 \end{array}\right)+\lambda \left(\begin{array}{ccc} 7 & 0 & 0\\ 0 & 30 & 0\\ 0 & 0 & 20 \end{array}\right)+\left(\begin{array}{ccc} -2 & 6 & 0\\ 2 & 16 & 3\\ 0 & 5 & 0 \end{array}\right). \end{array}$$

We use Method 1 to compute Example 8. We present the results of Example 8 with random initial point in Table 5.

## 5. Conclusion

In this paper, we reformulate the EiCP as a system of continuously differentiable equations and use the Levenberg-Marquardt method to solve them. The numerical experiments show that our method is a promising method for solving the EiCP. The numerical experiments of the extensions confirm the efficiency of our method.

## Figures and Tables

**Table 1:** 

Method 1	*λ*	*x*	SPA	*λ*	*x*
	4.99542	(0.24366,0.75633)^*T*^		5	(0.25,0.75)^*T*^
	7.00343	(0.50625,0.49373)^*T*^		⋯	⋯
	7.66150	(0.71184,0.28784)^*T*^		8	(1,0)^*T*^

**Table 2:** 

Method 1	*λ*	*x*	SPA	*λ*	*x*
	4.60705	(0.55617,0.44898,0.13390)^*T*^		4.1340	(0,1,0.2679)^*T*^
	4.18288	(0.08023,0.51776,0.38374)^*T*^		5	(0.3333,1,0)^*T*^
	5.03988	(0.14265,0.42924,0.42811)^*T*^		6	(0,0,1)^*T*^
	5.87521	(0.30666,0.27220,0.50890)^*T*^		8	(1,0,0)^*T*^
	6.00946	(0.12341,0.36685,0.50975)^*T*^		⋯	⋯
	7.01141	(0.35296,0.30395,0.38277)^*T*^		⋯	⋯
	8.00737	(0.40141,0.35921,0.23934)^*T*^		⋯	⋯
	9.36479	(0.47314,0.290185,0.23667)^*T*^		⋯	⋯
	9.99838	(0.57695,0.13243,0.29060)^*T*^		⋯	⋯

**Table 3:** 

Method 1	*λ*	*x*	SPA	*λ*	*x*
	26.28209	(0.32922,0.28109,0.00067,0.68363)^*T*^		100	(1,0,0,0)^*T*^
	26.41489	(0.34225,0.04798,0.30622,0.31649)^*T*^		158	(0,1,0,0)^*T*^
	28.71143	(0.59383,0.30244,0.01458,0.43984)^*T*^		⋯	⋯
	29.13415	(0.39357,0.16933,0.31781,0.12821)^*T*^		⋯	⋯
	32.60775	(0.35641,0.26564,0.17973,0.62135)^*T*^		⋯	⋯
	32.86388	(0.13771,0.33320,0.02167,0.50745)^*T*^		⋯	⋯
	37.57690	(0.30291,0.19045,0.21854,0.27608)^*T*^		⋯	⋯
	41.01635	(0.28247,0.19667,0.23447,0.26953)^*T*^		⋯	⋯
	46.46811	(0.34130,0.28631,0.18549,0.16868)^*T*^		⋯	⋯
	49.14424	(0.25544,0.18482,0.28418,0.27187)^*T*^		⋯	⋯
	66.96950	(0.47995,0.22175,0.00256,0.29497)^*T*^		⋯	⋯
	77.42278	(0.26550,0.28105,0.30904,0.14384)^*T*^		⋯	⋯
	77.45752	(0.21870,0.31154,0.24021,0.22741)^*T*^		⋯	⋯
	99.42333	(0.36284,0.32018,0.16593,0.13579)^*T*^		⋯	⋯
	99.99801	(0.67443,0.13866,0.00391,0.18299)^*T*^		⋯	⋯
	107.49890	(0.39982,0.33534,0.17631,0.08845)^*T*^		⋯	⋯
	127.38892	(0.32969,0.37065,0.18570,0.10872)^*T*^		⋯	⋯
	148.53897	(0.34127,0.45333,0.01989,0.17409)^*T*^		⋯	⋯
	157.99623	(0.28006,0.37495,0.14411,0.07701)^*T*^		⋯	⋯
	197.17149	(0.48407,0.60907,0.15118,0.18058)^*T*^		⋯	⋯
	204.57942	(0.55801,0.67908,0.17056,0.14984)^*T*^		⋯	⋯
	226.27891	(0.26236,0.32696,0.08010,0.01135)^*T*^		⋯	⋯
	231.92132	(0.26744,0.33127,0.04324,0.00457)^*T*^		⋯	⋯

**Table 4:** 

*λ*	*μ*	*x*	*y*
0.95803	0.96145	(0.69993,0.30006)^*T*^	(0.69782,0.30217)^*T*^
0.96205	0.97990	(0.69259,0.30740)^*T*^	(0.68925,0.31074)^*T*^
0.95574	0.94739	(0.48255,0.51744)^*T*^	(0.47451,0.52548)^*T*^
0.96069	0.93502	(0.37373,0.62626)^*T*^	(0.37670,0.62329)^*T*^
0.94582	1.04493	(0.69415,0.30584)^*T*^	(0.69598,0.30401)^*T*^
0.96200	1.03264	(0.46627,0.53372)^*T*^	(0.47934,0.52065)^*T*^
0.95517	1.06445	(0.35869,0.64130)^*T*^	(0.37196,0.62803)^*T*^
0.95744	1.05645	(0.39169,0.60830)^*T*^	(0.39707,0.60292)^*T*^

**Table 5:** 

*λ*	*x*
0.25315	(0.97503,0.01611,0.00884)^*T*^
−4.38990	(0.00000,1.00000,0.00000)^*T*^
−3.74482	(0.95986,0.02679,0.01334)^*T*^
−0.84644	(0.39691,0.42913,0.17392)^*T*^
−5.000000	(0.00000,1.00000,0.00000)^*T*^
−0.60280	(0.40400,0.37328,0.22271)^*T*^
−3.66880	(0.83349,0.10559,0.06090)^*T*^
−1.87686	(0.19023,0.25846,0.55130)^*T*^
−1.98258	(0.04642,0.06140,0.89217)^*T*^
−0.01062	(0.10208,0.03658,0.86133)^*T*^
